# Association between systemic inflammatory response index and left ventricular remodeling and systolic dysfunction in atrial fibrillation patients

**DOI:** 10.1186/s12872-023-03403-8

**Published:** 2023-07-28

**Authors:** Runze Chi, Xiaoli Shan, ChunPing Guan, Hao Yang, Xiangkun Wang, Bingong Li, Qing Zhang

**Affiliations:** 1 Qingdao Municipal Hospital, Qingdao University Affiliated Qingdao Municipal Hospital, 266011 Qingdao, Shandong China; 2 Weifang Medical University, 261000 Weifang, Shandong China; 3 Department of Cardiology, Qingdao Hospital, University of Health and Rehabilitation Sciences (Qingdao Municipal Hospital), 266011 Qingdao, Shandong China

**Keywords:** Atrial fibrillation, Left ventricular remodeling, Left ventricular systolic dysfunction, Systemic inflammatory response index

## Abstract

**Background:**

Cardiac remodeling and dysfunction can be caused by atrial fibrillation (AF). The aim of this research is to investigate the relationship between the systemic inflammatory response index (SIRI) and left ventricular (LV) remodeling and systolic function in individuals with AF.

**Methods:**

416 patients with AF who were admitted to the Second Department of Cardiology in the East Ward of the Qingdao Municipal Hospital between January 2020 and May 2022 were included in the present retrospective research. The relationship between SIRI and various cardiac parameters was analyzed. The patients’ left atrial (LA) enlargement and left ventricular (LV) hypertrophy and systolic dysfunction were evaluated. SIRI was calculated by the formula: neutrophil × monocyte/lymphocyte.

**Results:**

SIRI significantly correlated with LV end-diastolic diameter (LVDd), LV posterior wall thickness at end-diastole (LVPWTd), interventricular septal thickness at end-diastole (IVSTd), LV mass index (LVMI), LV ejection fraction (LVEF), LA diameter (LAD), C-reactive protein (CRP), and N-terminal pro-B-type natriuretic peptide (NT-proBNP) in patients with AF. In multivariate linear regression analyses, SIRI was discovered to be significantly related to LVMI (ln-transformed) (*p* = 0.025), LVEF (ln-transformed) (*p* = 0.005), and LAD (ln-transformed) (*p* = 0.007). In multivariate logistic regression, the highest quartile of SIRI (SIRI > 1.62) was significantly associated with LV hypertrophy (*p* = 0.026), impaired LV systolic function (*p* = 0.002), and LA enlargement (*p* = 0.025).

**Conclusions:**

SIRI was significantly associated with LV remodeling and systolic function impairment in patients with AF. SIRI may serve as a reliable and convenient inflammatory biomarker for detecting impaired cardiac structure and systolic function in patients with AF.

## Introduce

Atrial fibrillation (AF) has emerged as the predominant arrhythmia with a steady rise in incidence and prevalence over the past two decades, affecting a substantial proportion of the global population, estimated at approximately 37.5 billion as of 2017 [1]. The development of heart failure, a major complication of AF, poses significant challenges to patient quality of life and healthcare costs [2]. The early stages of AF can lead to heart remodeling and dysfunction, culminating in heart failure [3]. Timely detection and intervention of such structural and functional cardiac impairments are therefore crucial to impede the progression towards heart failure.

Recent evidence has linked AF to endothelial dysfunction and inflammatory response in heart tissue triggered by systemic inflammation, resulting in a pro-inflammatory environment, which in turn contributes to cardiac remodeling and dysfunction [3]. In this context, the systemic inflammatory response index (SIRI), a new inflammatory biomarker, has been shown to have predictive value in various disease states, including tumors, acute ischemic stroke, and cardiovascular disease risk [4–7]. SIRI, calculated as the neutrophil × monocyte/lymphocyte ratio, serves as a comprehensive indicator of chronic low-grade inflammation within the patient’s body. Notably, a study by Dziedzic revealed elevated SIRI levels among women diagnosed with acute coronary syndrome compared to those with stable coronary artery disease [8]. Additionally, in the investigation conducted by Fan, a significant correlation was observed between higher SIRI values and the occurrence of major cardiovascular events following percutaneous coronary intervention in patients with acute coronary syndromes [9]. It has also been shown that there is a significant association between SIRI and the occurrence of new-onset atrial fibrillation in patients with ST-segment elevation myocardial infarction undergoing percutaneous coronary intervention [10]. However, the association between SIRI and left ventricular (LV) remodeling and systolic function in AF patients is yet to be fully elucidated. The present study aims to examine the relationship between SIRI and LV remodeling and systolic function in patients with AF.

## Methods

### Study population

**Fig. 1:**
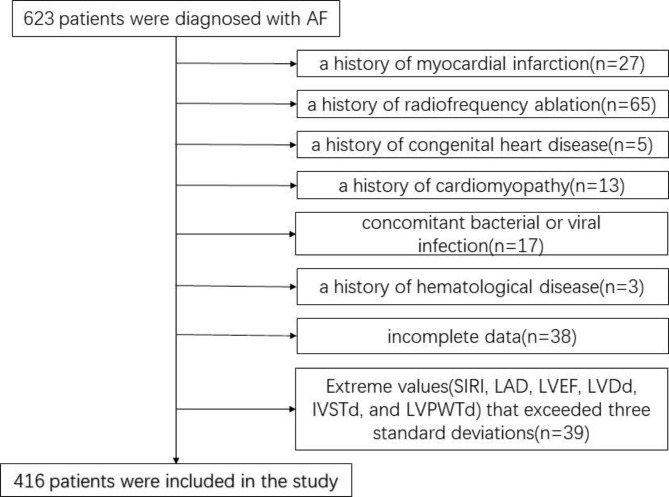
Flowchart of the study population. SIRI, systemic inflammatory response index; SEM, the standard error of the mean; LAD, left atrial diameter (mm); LVEF, LV ejection fraction (%); LVDd, LV diastolic diameter (mm); LVPWTd, LV posterior wall thickness (mm); IVSTd, interventricular septal wall thickness (mm)

Retrospectively, data on 416 individuals who matched the inclusion were enrolled and examined from January 2020 to May 2022 at the Second Department of Cardiology in the East Ward of Qingdao Municipal Hospital. The inclusion criteria required that all patients were diagnosed with AF by electrocardiogram. The criteria for exclusion were: (1) a history of myocardial infarction; (2) a history of radiofrequency ablation; (3) a history of congenital heart disease; (4) a history of cardiomyopathy; (5) concomitant bacterial or viral infection; (6) a history of hematological disease; (7) a history of rheumatological diseases; (8) incomplete data. The flowchart depicting the process of patient selection is presented in Fig. 1. All clinical information used in the current research was retrospectively gathered from the medical records of each patient. Due to the study’s retrospectively design, informed patient assent was not required.

### Data collection

The baseline characteristics of patients were obtained from the inpatient record system, which included gender, age, height, weight, history of type 2 diabetes mellitus, history of hypertension, history of coronary heart disease and medication history. Laboratory data was collected on the second day after admission from fasting venous blood samples, which included C-reactive protein (CRP), cardiac troponin I (cTN-I), N terminal pro B type natriuretic peptide (NT-proBNP), the contents of triglyceride (TG), total cholesterol (TC), low-density lipoprotein cholesterol (LDL-C) and high-density lipoprotein cholesterol (HDL-C). The echocardiographic parameters recorded were left atrial diameter (LAD), LV ejection fraction (LVEF), LV diastolic diameter (LVDd), the diastolic interventricular septal wall thickness (IVSTd), and the diastolic LV posterior wall thickness (LVPWTd). LA enlargement was defined as LAD > 40 mm. LV hypertrophy (LVH) was determined using LV mass index (LVMI) > 95 g/m² for female patients and LVMI > 115 g/m² for male patients, calculated as LVMI = left ventricular mass (LVM) / body surface area (BSA); LVM = 0.8 × 1.04 [(LVDd + LVPWTd + IVSTd)^3^ − (LVDd)^3^] + 0.6; BSA = 0.0061 × height (cm) + 0.0128 × weight (kg) – 0.1529; Relative wall thickness(RWT) = (2 × LVPWTd)/(LVDd)[11]. LV systolic dysfunction was defined as an ejection fraction of < 50%. The SIRI formula was used to calculate SIRI, which was determined as SIRI = N×M/L, where N were defined as peripheral neutrophil counts, M were defined as monocyte counts, and L were defined as lymphocyte counts.

### Statistical analysis

Using SPSS version 27.0, data for this investigation were analyzed. GraphPad Prism version 9.5.1 was utilized for graphic plotting. Extreme values (SIRI, LAD, LVEF, LVDd, LVPWTd and IVSTd) that exceeded three standard deviations from the mean were excluded before SIRI values were grouped. SIRI levels were categorized into quartiles, with Q1 representing the first quartile, Q2 representing the second quartile, Q3 representing the third quartile, and Q4 representing the fourth quartile. The Q-Q diagram was used to determine whether or not the continuous data distribution was normal. To compare continuous variables with normal distributions that were provided as mean ± standard deviation (SD), one-way analysis of variance was used. By showing variables that did not follow a normal distribution as median and interquartile range, the Kruskal-Wallis test was used to analyze variables. The Pearson χ2 test was used to compare the baseline characteristics of categorical variables, which were given as numbers and percentages. To examine the relationship between SIRI and LAD, LVEF, LVDd, LVPWTd, IVSTd, LVMI, RWT, CRP and NT-proBNP, Spearman’s rank correlation was used. SIRI, LAD, LVEF, LVDd, LVPWTd, IVSTd, LVMI, and RWT were found to have a nonnormal distribution and were converted to a natural log transformation (ln-transformed) to make it more close to a normal distribution, referred as ln-transformed SIRI, ln-transformed LAD, ln-transformed LVEF, ln-transformed LVDd, ln-transformed LVPWTd, ln-transformed IVSTd, ln-transformed LVMI, and ln-transformed RWT. Linear regression analysis was investigated the connection between SIRI and the following echocardiographic measurements: LAD (ln-transformed), LVEF (ln-transformed), LVDd (ln-transformed), LVPWTd (ln-transformed), IVSTd (ln-transformed), LVMI (ln-transformed), and RWT (ln-transformed). The SIRI was set as an independent variable, and echocardiographic parameters were set as dependent variables, and their correlation was analyzed using univariate linear regression (Model 1). Two multivariate linear regression models (Model 2 and Model 3) were used after adjusting for confounding factors. Age, sex, BMI, smoking history, and alcohol use were all taken into account in Model 2, while Type 2 diabetes mellitus, Hypertension, Coronary heart disease, and type of AF were all taken into account additionally in Model 3. Univariate and multivariate logistic regression analyses were used to explore the relationship between SIRI levels and the prevalence of LVH, LV impaired systolic function, and LA enlargement, using the above three models.

## Results

### Characteristic of patients’

The study included 416 patients, with a mean age of 68.71 ± 12.70 and a gender split of 194 males and 222 females. Figure 1 illustrates the patient distribution. Table 1 lists the patients’ baseline characteristics. Results indicate that patients with greater SIRI levels had older age, higher proportion of males, and increased incidence of heart failure and persistent AF. These groups’ variations had been found to be statistically meaningful (*p* < 0.05, Table 1). Conversely, no statistically meaningful variation was present between the SIRI level groups with regards to height, weight, BMI, history of type 2 diabetes mellitus, hypertension, coronary heart disease, smoking or drinking. The medication groups ACEI/ARB/ARNI, CCB, β-blocker, diuretic, and Lipid-lowering drugs also did not exhibit any significant differences. However, laboratory data demonstrated significant differences (*p* < 0.05, Table 1) in NT-proBNP, TC, TG, LDL, WBC count, neutrophil count, monocyte count, lymphocyte count and CRP, while HDL and platelet count did not exhibit any statistical significance.

**Table 1:** Baseline characteristics of patients according to different SIRI quartiles

Variables	SIRI				p-value
	1	2	3	4	
Quartile range	≤ 0.72	0.72–1.06	1.07–1.62	> 1.62	-
n	104	104.00	104	104	-
Age, years	66.14 ± 12.28	67.10 ± 13.95	69.43 ± 12.94	72.17 ± 10.72	0.003
Male, n (%)	36(34.6%)	59(56.7%)	61(58.7%)	66(63.5%)	< 0.001
height(cm)	165.03 ± 8.42	166.77 ± 8.22	166.76 ± 8.75	167.74 ± 7.70	0.124
weight(kg)	72.20 ± 12.09	72.57 ± 12.42	72.82 ± 11.80	73.19 ± 11.18	0.944
BMI	26.48 ± 3.74	25.97 ± 3.17	26.16 ± 3.78	26.01 ± 3.62	0.733
persistent AF, n (%)	38(36.5%)	49(47.1%)	53(51.0%)	59(56.7%)	0.029
Type 2 diabetes mellitus, n (%)	23(22.1%)	28(26.9%)	32(30.8%)	34(32.7%)	0.339
Hypertension, n (%)	61(58.7%)	58(55.8%)	57(54.8%)	71(68.3%)	0.180
Coronary heart disease, n (%)	46(44.2%)	47(45.2%)	48(46.2%)	52(50.0%)	0.849
Heart failure, n (%)	9(8.7%)	12(11.5%)	16(15.4%)	30(28.8%)	< 0.001
Smoking, n (%)	18(17.3%)	24(23.1%)	22(21.2%)	27(26.0%)	0.493
Drinking, n (%)	12(11.5%)	18(17.3%)	18(17.3%)	22(21.2%)	0.320
Medications					
ACEI/ARB/ARNI, n (%)	33(31.7%)	36(34.6%)	31(29.8%)	43(41.3%)	0.317
β blocker, n (%)	28(26.9%)	32(30.8%)	31(29.8%)	25(24.0%)	0.697
CCB, n (%)	26(25.0%)	20(19.2%)	25(24.0%)	36(34.6%)	0.079
Diuretic, n (%)	13(12.5%)	12(11.5%)	12(11.5%)	17(16.3%)	0.695
Lipid-lowering drugs, n (%)	22(21.2%)	14(13.5%)	23(22.1%)	27(26.0%)	0.156
NT-proBNP, pg/ml	373.74(62.45,846.63)	475.4(221.24,1125.71)	506.37(211.56,1405.75)	968.46(325,50,2555.76)	< 0.001
TC, mmol/L	4.27(3.39,5.10)	4.30(3.53,5.11)	3.73(3.10,4.64)	3.55(3.00,4.49)	< 0.001
TG, mmol/L	1.25(0.93,1.69)	1.21(0.92,1.65)	1.06(0.82,1.50)	1.03(0.77,1.41)	0.008
HDL, mmol/L	1.05(0.93,1.25)	1.07(0.95,1.21)	1.03(0.91,1.21)	1.05(0.84,1.18)	0.381
LDL, mmol/L	2.53(1.82,3.20)	2.61(2.09,3.16)	2.22(1.67,2.82)	2.03(1.62,2.71)	< 0.001
WBC count, ×10^9^/L	5.26(4.56,6.56)	6.09(5.33,6.99)	6.62(5.65,7.60)	7.65(6.63,9.08)	< 0.001
Neutrophils count, ×10^9^/L	2.77(2.26,3.43)	3.63(3.05,4.26)	4.19(3.70,4.86)	5.68(4.59,6.53)	< 0.001
Monocyte count, ×10^9^/L	0.36(0.29,0.43)	0.44(0.38,0.52)	0.51(0.41,0.63)	0.61(0.48,0.74)	< 0.001
Lymphocyte count, ×10^9^/L	1.98(1.68,2.42)	1.85(1.51,2.22)	1.55(1.25,2.12)	1.37(1.07,1.78)	< 0.001
Platelet count, ×10^9^/L	205.50(166.00,238.75)	201.00(161.75,236.75)	206.00(163.75,242.50)	200.50(164.25,238.750)	0.912
CRP, mg/L,	0.66(0.50,2.17)	0.52(0.5,1.93)	0.50(0.50,2.43)	1.71(0.50,7.22)	0.018

Note: All data are presented as mean ± SD and median (25%,75%) or n (%)

Abbreviations: SIRI, systemic inflammatory response index; ACEI, angiotensin-converting enzyme inhibitors; ARB, angiotensin receptor blocker; BMI, body mass index; CCB, calcium channel blocker; TC, total cholesterol; TG, triglyceride; HDL‐C, high‐density lipoprotein cholesterol; LDL‐C, low‐density lipoprotein cholesterol; CRP, C-reactive protein

### Association between SIRI levels and echocardiographic parameters

**Fig. 2:**
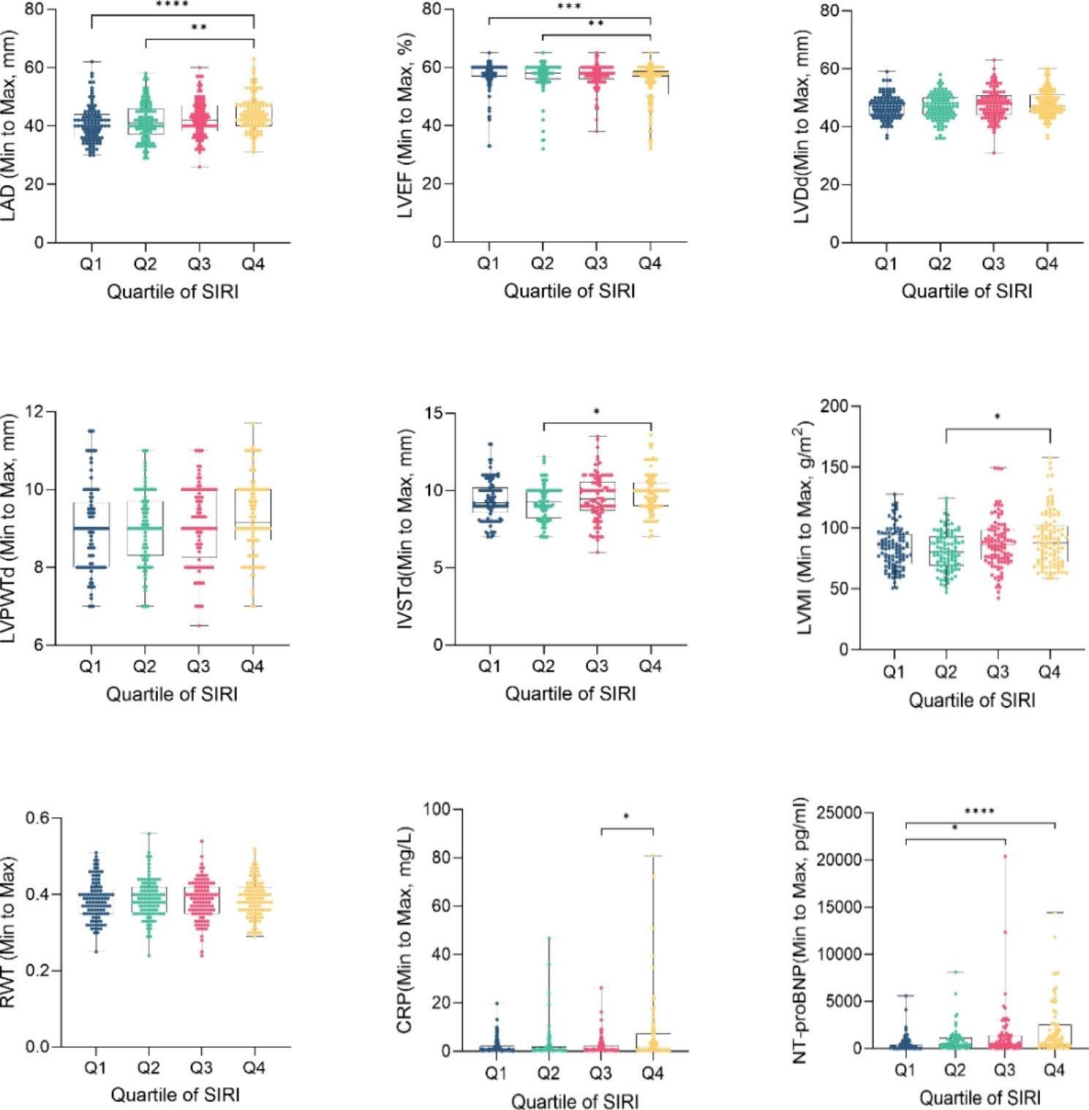
Left cardiac structure, systolic function, CRP and NT-proBNP of patients grouped by quartile of SIRI levels. SIRI, systemic inflammatory response index; SEM, the standard error of the mean; LAD, left atrial diameter (mm); LVEF, LV ejection fraction (%); LVDd, LV diastolic diameter (mm); LVPWTd, LV posterior wall thickness (mm); IVSTd, interventricular septal wall thickness (mm); LVMI, left ventricular mass index (g/m2); RWT, relative wall thickness; CRP, C-reactive protein (mg/ml); NT-proBNP, N-terminal pro-brain natriuretic peptide (pg/ml). *Adjusted by Bonferroni method < 0.05(adjusted *p*); **<0.01(adjusted *p*); ***<0.001(adjusted *p*); ****<0.0001(adjusted *p*)

**Fig. 3:**
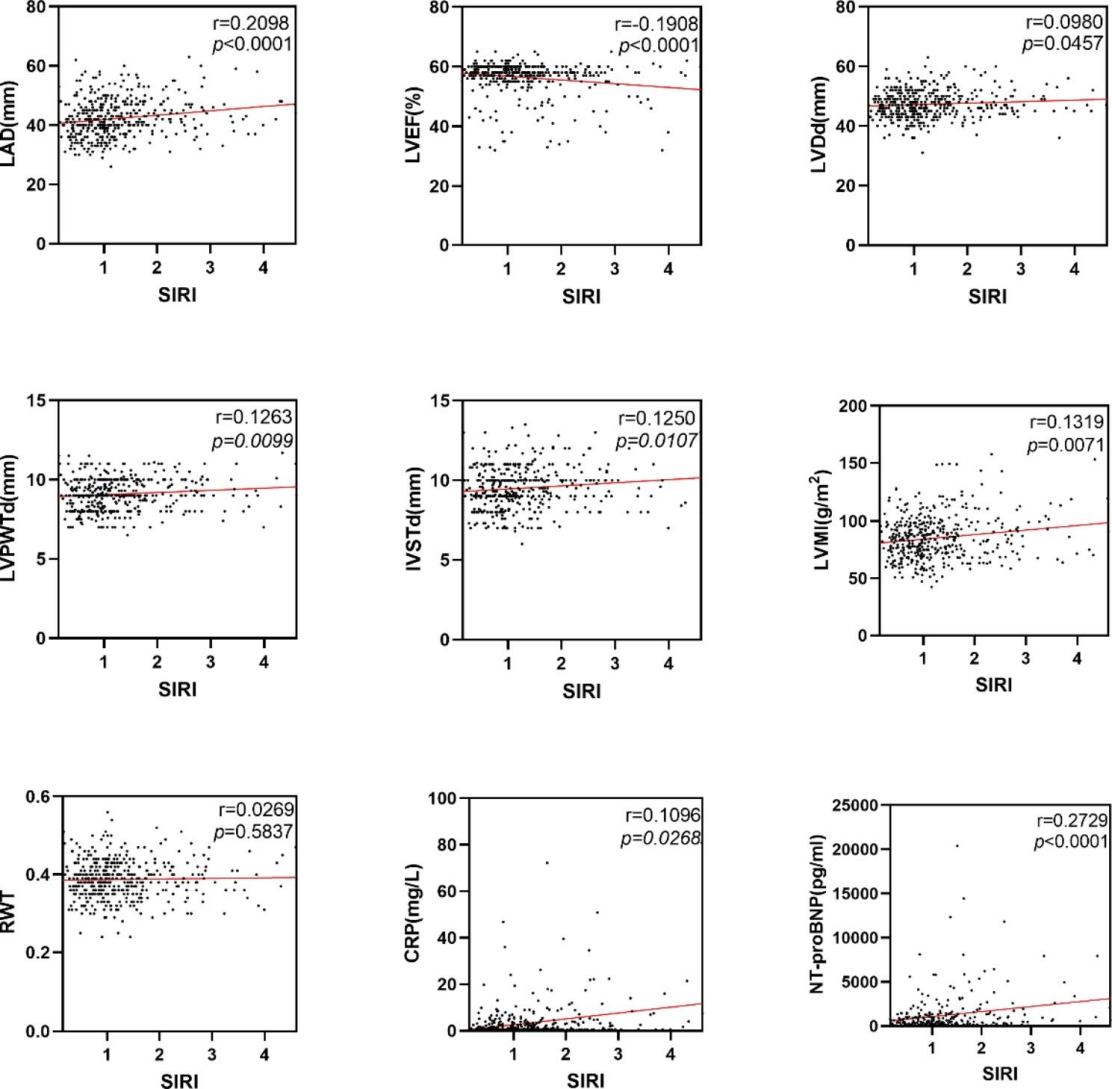
Correlation between left cardiac structure, systolic function, CRP and NT-proBNP and SIRI. SIRI, systemic inflammatory response index; LAD, left atrial diameter; LVEF, LV ejection fraction; LVDd, LV diastolic diameter; LVPWTd, LV posterior wall thickness; IVSTd, interventricular septal wall thickness; LVMI, left ventricular mass index; RWT, relative wall thickness; CRP, C-reactive protein; NT-proBNP, N-terminal pro-brain natriuretic peptide

The present study investigated the association between SIRI and left cardiac structural and functional parameters, as well as laboratory data markers, in patients with AF. Echocardiography was used to measure LAD, LVPWTd, LVEF, LVDd, IVSTd, LVMI, and RWT in patients across SIRI quartiles. The results showed that LAD (Q4 vs. Q2, adjusted *p* < 0.01; Q4 vs. Q1, adjusted *p* < 0.0001), IVSTd (Q4 VS Q2, adjusted *p* < 0.05) and LVMI (Q4 vs. Q2, adjusted *p* < 0.05) increased significantly as SIRI quartiles increased, while LVEF decreased significantly (Q4 vs. Q2, adjusted *p* < 0.01; Q4 vs. Q1, adjusted *p* < 0.001). There was no significant difference in LVDd, LVPWd, and RWT between SIRI quartiles. Additionally, laboratory data revealed that CRP and NT-proBNP increased as SIRI quartile level increased (Q4 vs. Q3, adjusted *p* < 0.05 for CRP; Q4 vs. Q1, adjusted *p* < 0.0001, Q3 vs. Q2, adjusted *p* < 0.05 for NT-proBNP) (Fig. 2). These findings suggest a significant correlation between SIRI and LAD (r = 0.2098, *p* < 0.0001), LVEF (r=-0.1908, *p* < 0.0001), LVDd (r = 0.0980, *p* = 0.0457), LVPWTd (r = 0.1263, *p* = 0.0099), IVSTd (r = 0.1250, *p* = 0.0107), LVMI (r = 0.1319, *p* = 0.0071), CRP (r = 0.1096, *p* = 0.0286), and NT-proBNP (r = 0.2729, *p* < 0.0001) in patients with AF (Fig. 3).

### Univariate and multivariate linear regression analyses

Univariate linear regression (Model 1) analysis performed that SIRI was positively related to IVSTd (ln-transformed) (*p* = 0.020), LVPWTd (ln-transformed) (*p* = 0.025), LVMI (ln-transformed) (*p* = 0.001), LVEF (ln-transformed) (*p* < 0.001) and LAD (ln-transformed) (*p* < 0.001) (Table 2). After accounting for factors such as age, gender, BMI, smoking and alcohol habits (Model 2), SIR remained positively associated with LVMI (ln-transformed) (*p* = 0.017), LVEF (ln-transformed) (*p* = 0.001) and LAD (ln-transformed) (*p* < 0.001) (Table 2). Further adjusting for type 2 diabetes mellitus history, hypertension history and type of AF (Model 3), SIRI remained positively associated with LVMI (ln-transformed) (*p* = 0.025), LVEF (ln-transformed) (*p* = 0.005) and LAD (ln-transformed) (*p* = 0.007) (Table 2).

**Table 2:** Linear regression analysis assessing the relationships of SIRI levels with left cardiac structural and functional parameters

Echocardiographic parameters	model1			model2			model3		
	*β*	SE	*p*-value	*β*	SE	*p*-value	*β*	SE	*p*-value
LV structure									
LVDd (ln-transformed)	0.011	0.006	0.067	0.009	0.006	0.140	0.007	0.006	0.200
IVSTd (ln-transformed)	0.019	0.008	0.020	0.015	0.008	0.064	0.016	0.008	0.053
LVPWTd (ln-transformed)	0.015	0.007	0.025	0.007	0.006	0.263	0.006	0.006	0.340
RWT (ln-transformed)	0.004	0.008	0.586	-0.001	0.008	0.867	-0.001	0.008	0.872
LVMI (ln-transformed)	0.042	0.013	0.001	0.032	0.013	0.017	0.030	0.013	0.025
LV systolic function									
LVEF (ln-transformed)	0.100	0.029	< 0.001	0.099	0.030	0.001	0.080	0.028	0.005
LA structure									
LAD (ln-transformed)	0.035	0.009	< 0.001	0.030	0.009	< 0.001	0.021	0.008	0.007

Abbreviations: SIRI, systemic inflammatory response index; LV, left ventricular; LAD, left atrial diameter; LVEF, LV ejection fraction; LVDd, LV diastolic diameter; LVPWTd, LV posterior wall thickness; IVSTd, interventricular septal wall thickness; LVMI, left ventricular mass index; RWT, relative wall thickness

### Univariate and multivariate logistic regression

In the 416 AF patients, LVH, impaired LV systolic function and LAD enlargement were detected in 72 (17.3%), 41 (9.9%) and 241 (57.9%). In univariate logistic regression (Model 1), the highest SIRI quartile (SIRI > 1.62) was significantly associated with impaired LV systolic function (OR = 3.72, 95%CI = 1.51–9.14, *p* = 0.004) and LA enlargement (OR = 3.20,95%CI = 1.79–5.74, *p* < 0.001) (Table 3). In multivariate logistic regression (Model 2 and Model 3), the highest SIRI quartile (SIRI > 1.62 ) was significantly associated with LVH (OR = 2.58, 95%CI = 1.21–5.52, *p* = 0.014 for Model 2; OR = 2.39, 95%CI = 1.11–5.13, *p* = 0.026 for Model 3), impaired LV systolic function (OR = 5.61, 95%CI = 2.15–14.60), *p* < 0.001 for Model 2; OR = 4.78, 95%CI = 1.79–12.78, *p* = 0.002 for Model 3) and LA enlargement (OR = 2.60, 95%CI = 1.39–4.88, *p* = 0.003 for Model 2; OR = 2.20, 95%CI = 1.10–4.39, *p* = 0.025 for Model 3) (Table 3).

**Table 3:** Logistic regression analysis assessing the relationships of SIRI with LV hypertrophy, LV impaired systolic function and left atrial enlargement

	model1		model2		model3	
	OR (95%CI)	*p*-value	OR (95%CI)	*p*-value	OR (95%CI)	*p*-value
LV hypertrophy						
Q1	1(Reference)	-	1(Reference)	-	1(Reference)	-
Q2	0.67(0.30–1.48)	0.319	0.85(0.37–1.96)	0.700	0.82(0.35–1.91)	0.645
Q3	1.00(0.48–2.09)	1.000	1.30(0.59–2.86)	0.510	1.20(0.54–2.68)	0.651
Q4	1.71(0.86–3.38)	0.126	2.58(1.21–5.52)	0.014	2.39(1.11–5.13)	0.026
LV impaired systolic function						
Q1	1(Reference)		1(Reference)		1(Reference)	-
Q2	0.85(0.28–2.62)	0.775	1.02(0.32–3.19)	0.980	0.92(0.28–2.97)	0.886
Q3	0.85(0.28–2.62)	0.775	1.14(0.36–3.59)	0.826	0.92(0.29–2.99)	0.895
Q4	3.72(1.51–9.14)	0.004	5.61(2.15–14.60)	< 0.001	4.78(1.79–12.78)	0.002
Left atrial enlargement						
Q1	1(Reference)	-	1(Reference)	-	1(Reference)	-
Q2	1.21(0.70–2.09)	0.488	1.14(0.64–2.03)	0.662	0.94(0.49–1.80)	0.859
Q3	1.59(0.92–2.76)	0.096	1.39(0.77–2.50)	0.272	1.13(0.59–2.18)	0.711
Q4	3.20(1.79–5.74)	< 0.001	2.60(1.39–4.88)	0.003	2.20(1.10–4.39)	0.025

Abbreviations: SIRI, systemic inflammatory response index; LV, left ventricular

## Discussion

The results of our study revealed a significant association between SIRI levels and cardiac structural and systolic functional impairment in patients with AF. Echocardiography data were analyzed and revealed that SIRI levels were independently associated with greater LAD, LVPWTd, LVMI, and IVSTd, as well as lower LVEF, but no correlation was found with LVDd and RWT. The laboratory results also showed that higher NT-proBNP and CRP levels were independently correlated with higher SIRI levels. Furthermore, LVH, impaired LV systolic function, and left atrial enlargement were all strongly linked with increased SIRI levels.

Numerous comorbidities, including as heart failure, myocardial infarction, chronic renal disease, venous thromboembolism, stroke, dementia, and cancer, have been related to AF [12]. Heart remodeling and dysfunction, which are characterized by changes in genetic expression, molecular, cellular, and interstitial components and clinically manifest as changes in the size, shape, and function of the heart as a result of cardiac damage, are principally responsible for the development of heart failure [13]. LV remodeling is a common target organ lesion in hypertensive patients. In patients with hypertension, elevated blood pressure occurs before the onset of LVMI and RWT, with hemodynamic properties serving as the primary cause of cardiac remodeling [14]. Patients with LV remodeling have a higher rate of AF, with AF promoting further development of cardiac remodeling [15, 16]. In a retrospective examination of data from 175 individuals with paroxysmal AF and 175 controls, Noirclerc et al. discovered that individuals with paroxysmal AF had larger left ventricular mass [16]. The major treatment for sinus rhythm restoration and maintenance in AF patients is catheter ablation. Studies have shown that catheter ablation can also reverse LV geometric remodeling. A retrospective study by Okada et al. investigated 376 cases of AF in individuals with LV systolic dysfunction following catheter ablation, revealing that 306 patients developed reverse LV remodeling (LV end-systolic volume of ≥ 15%) after 3 months of follow-up [17]. LV remodeling has been clinically correlated with coronary atherosclerosis, obesity, age, sex, and ejection fraction without structural heart disease in patients [18–20]. Atrial systolic function loss and irregular heart rate significantly increase hemodynamic impairment. When paroxysmal atrial fibrillation patients undergo catheter ablation, irregular atrial ventricular pacing mimicking atrial fibrillation led to impaired left ventricular systolic and diastolic function [21].

Cardiac remodeling is significantly influenced by inflammation, and systemic inflammation can be determined by measuring the white blood cell count, a laboratory test typically performed on every patient upon admission [22, 23]. The neutrophil to lymphocyte ratio (NLR) and monocyte to lymphocyte ratio (MLR) have surfaced as useful inflammatory markers in recent years that can be easily calculated using the white blood cell count. A retrospective and cross-sectional investigation of 386 patients with hypertension conducted by YU et al. reported that NLR was found to be significantly higher in individuals with LV hypertrophy [24]. Similarly, MLR was shown to be a predictor of the severity of coronary lesion and atherosclerosis [25]. This study introduces a novel inflammatory marker, SIRI, which is a composite index that calculates neutrophils, lymphocytes, and monocytes, and demonstrates superior sensitivity and specificity in predicting cardiovascular events and all-cause mortality when compared to NLR and MLR [4, 6, 7].

In our investigation, we observed a significant positive correlation between the levels of SIRI and CRP in patients diagnosed with atrial fibrillation. Importantly, these findings align with prior research conducted on patients affected by rheumatoid arthritis and diabetes, further supporting the consistency of such associations across different patient populations [26, 27]. CRP is the most commonly used marker to evaluate systemic inflammation in clinical practice. Infection, tissue injury, myocardial infarction and inflammatory diseases can lead to its increase. Furthermore, this study presents novel findings indicating a positive association between elevated SIRI levels and increased NT-proBNP concentrations in patients diagnosed with AF. NT-proBNP, which represents the N-terminal fragment of pro-brain natriuretic peptide, is released by cardiomyocytes in response to diverse stimuli, including myocardial pressure, cardiac volume load, and myocardial cell tension. Its physiological functions encompass vasodilation, natriuresis, and diuresis. Notably, previous investigations have suggested a potential anti-inflammatory role of natriuretic peptides [28]. For instance, the activation of cytokines can be inhibited through the stimulation of natriuretic peptide receptors found on macrophages, dendritic cells, and T cells [29–31]. As natriuretic peptides function as hormones regulated by a feedback loop, their anti-inflammatory effects primarily arise from the induction of natriuretic peptide release in response to inflammation [28]. However, it is crucial to note that the validity of these findings warrants further verification. It is also worth considering that the inclusion of a substantial number of abnormal NT-proBNP and CRP values within the study population may have an impact on the statistical results, underscoring the need for cautious interpretation.

Interestingly, notable statistically significant differences in lipid profiles were observed within the baseline data when grouped according to SIRI quartiles. Specifically, higher SIRI levels were associated with lower TC, TG, and LDL levels. The impact of inflammation on lipid parameters such as TG, TC, HDL, and LDL has been extensively described in various studies. For instance, patients with sepsis exhibit decreased serum concentrations of TC and HDL cholesterol, whereas triglyceride levels are increased [32]. Likewise, individuals with active rheumatoid arthritis tend to have lower levels of TC, HDL, and LDL compared to those without active disease [33]. It is worth noting that statins are the most commonly utilized lipid-lowering medications, possessing anti-inflammatory properties that affect the adhesion and migration of inflammatory cells, reduce the expression of chemokines such as monocyte-chemoattractant protein-1 and IL-8, and diminish the expression of Toll-like receptors on immune cells [34]. In the context of this study, TG levels displayed a gradual decrease with increasing SIRI levels in patients with atrial fibrillation. However, while TC and LDL levels in the second SIRI quartile were higher than those in the first quartile, an overall downward trend was still observed. HDL levels did not exhibit statistically significant differences across the four SIRI quartiles.

In our research we observe that Table 1 shows the increasing proportion of men according to the SIRI quantile. Previous studies have shown similar trends. The observed gender differences in the baseline data of SIRI quartiles could potentially be attributed to underlying mechanisms. Sex hormones, such as estrogen and testosterone, are known to modulate the inflammatory response. Estrogen exerts its anti-inflammatory effects through various mechanisms, including direct antioxidant properties, nitric oxide production, prevention of vascular cell death, inhibition of cytokines, and modulation of the renin-angiotensin system [35]. On the other hand, testosterone may exhibit both pro-inflammatory and anti-inflammatory effects. The anti-inflammatory effect of testosterone primarily stems from its capacity to decrease inflammatory cytokine levels, and it has been observed that testosterone deficiency is associated with an elevation in inflammatory cytokines. Additionally, testosterone can regulate cytokine release from immune cells and adipose tissue, contributing to its anti-inflammatory properties [36]. However, it is important to note that testosterone can also exert pro-inflammatory effects by promoting macrophages to release higher levels of TNFα and nitric oxide, as well as increasing the expression of TNF-α and IL-6 [37].

In this study, the highest SIRI values were linked to an increased chance of LAD enlargement, LVH, and poor LV systolic function. In Model 1 of this study, when comparing the first quantile as the reference, a significant association was observed only between the fourth quantile SIRI (SIRI > 1.62) and impaired left ventricular systolic function as well as LA enlargement. Additionally, SIRI levels displayed positive correlations with IVSTd (ln-transformed), LVPWTd (ln-transformed), LVMI (ln-transformed), LVEF (ln-transformed), and LAD (ln-transformed). In models 2 and 3, a significant association was found only between the fourth quantile SIRI (SIRI > 1.62) and LVH, impaired left ventricular systolic function, and LA enlargement when compared with the first quantile. Additionally, SIRI levels displayed positive correlations with LVMI (ln-transformed), LVEF (ln-transformed), and LAD (ln-transformed). Previously, only patients with a left ventricular ejection fraction of 40% or less were considered to have heart failure (heart failure with reduced ejection fraction, HFrEF), but subsequent evidence has shown that patients with a left ventricular ejection fraction of 41 to 49% (heart failure with mid-range ejection fraction, HFmrEF) have similar characteristics to HFrEF [38, 39]. Among structural/functional myocardial abnormalities, mild systolic dysfunction (LVEF between 40 and 53%) has received much attention, with patients with left ventricular systolic dysfunction having a higher risk of HF and death over a median follow-up of 5.7 years compared with patients with normal left ventricular systolic function [40]. Left ventricular systolic dysfunction was associated with a high risk of progression to congestive heart failure and death, independent of baseline cardiovascular risk factors [41, 42]. Thus, the term HF with mid-range ejection fraction was changed to heart failure with mildly reduced ejection fraction (HFmrEF) [43–47]. This study referred to other studies, which also defined patients with a left ventricular ejection fraction of less than 50% as having left ventricular systolic dysfunction, so that patients with a left ventricular ejection fraction of 41 to 49% were equally recognized and valued as those with a left ventricular ejection fraction of 40% or less [46–50]. Patients having a history of myocardial infarction as well as those with bacterial and viral infections were excluded in order to prevent patients from confusing inflammatory responses and cardiac remodeling following myocardial infarction. Inflammatory responses to cardiac remodeling involve neutrophils, lymphocytes, and monocytes, which are involved in the three phases of myocardial remodeling and cardiac repair: inflammatory phase, proliferative and reparative phase, and maturation phase [51]. Additionally, oxidative stress, Ca^2+^/calmodulin-dependent protein kinase IIδc oxidation, and Ca^2+^/calmodulin-dependent protein kinase IIδc activity in the left ventricular myocardium contribute to adverse remodeling of the AF LV [52].

This study’s limitations encompass the enrollment of patients receiving inpatient care and the single-center, small-scale design. Additionally, the employment of SIRI as a dynamic biomarker for computing leukocyte counts is susceptible to variations stemming from inaccuracies in blood sample collection, preservation, and measurement. Furthermore, this research is of a retrospective, cross-sectional case-control nature; future prospective studies are necessary to corroborate the findings.

## Conclusion

In summary, our investigation had evidenced a substantial correlation between elevated SIRI and LV remodeling and systolic functional impairment in patients with AF. The observed association primarily manifested as LVH, LV systolic dysfunction, and left atrial enlargement. Our findings suggested that SIRI may serve as a dependable and convenient inflammatory biomarker for detecting cardiac structural damage and systolic functional impairment in AF patients. The incorporation of SIRI as an inflammatory marker in the evaluation and care of individuals with AF holds promise for risk stratification, treatment decision-making, and patient surveillance. This approach enables the implementation of a more individualized management approach for patients with AF.

## Data Availability

Raw data is available from the corresponding author.
